# Genomic Epidemiology of SARS-CoV-2 in Western Burkina Faso, West Africa

**DOI:** 10.3390/v14122788

**Published:** 2022-12-14

**Authors:** Yacouba Sawadogo, Lokman Galal, Essia Belarbi, Arsène Zongo, Grit Schubert, Fabian Leendertz, Abdoulie Kanteh, Abdul Karim Sesay, Annette Erhart, Anne-Laure Bañuls, Zékiba Tarnagda, Sylvain Godreuil, Halidou Tinto, Abdoul-Salam Ouedraogo

**Affiliations:** 1 Departement of Bacteriology and Virology, Souro Sanou University Hospital, Bobo Dioulasso 01 BP 676, Burkina Faso; 2 Laboratory of Emerging and Re-emerging Pathogens, School of Health Sciences Nazi Boni University, Bobo Dioulasso 01 BP 1091, Burkina Faso; 3 Laboratoire de Bactériologie, Centre Hospitalier Universitaire de Montpellier, 34295 Montpellier, France; 4 Maladies Infectieuses et Vecteurs: Ecologie, Génétique, Evolution et Contrôle (MIVEGEC), Université de Montpellier—Institut de Recherche pour le Développement (IRD)—Centre National de la Recherche Scientifique (CNRS), 34394 Montpellier, France; 5 Robert Koch Institute, 13353 Berlin, Germany; 6 Muraz Center, Bobo Dioulasso 01 BP 390, Burkina Faso; 7 Helmholtz Institut für One Health, 17489 Greifswald, Germany; 8 Genomics Core Facility, Medical Research Council Unit the Gambia (MRCG), London School of Hygiene and Tropical Medicine, Fajara P.O. Box 273, The Gambia; 9 Disease Control and Elimination Theme, Medical Research Council Unit the Gambia (MRCG), London School of Hygiene and Tropical Medicine, Fajara P.O. Box 273, The Gambia; 10 Jeune Equipe Associée (JEAI), Institut de Recherche pour le Développement (IRD), Résistances Antimicrobiennes au Burkina Faso (FASORAM), 34394 Montpellier, France; 11 Laboratoire National de Référence-Grippe (LNR-G), Institut de Recherche en Sciences de la Santé, Ouagadougou 03 BP 7192, Burkina Faso; 12 Centre National de la Recherche Scientifique et Technologique/Institut de Recherche en Sciences de la Santé (CNRST/IRSS), Nanoro BP 18, Burkina Faso

**Keywords:** COVID-19, SARS-CoV-2, whole genome sequencing, genomic epidemiology, West Africa, Burkina Faso

## Abstract

Background: After its initial detection in Wuhan, China, in December 2019, SARS-CoV-2 has spread rapidly, causing successive epidemic waves worldwide. This study aims to provide a genomic epidemiology of SARS-CoV-2 in Burkina Faso. Methods: Three hundred and seventy-seven SARS-CoV-2 genomes obtained from PCR-positive nasopharyngeal samples (PCR cycle threshold score < 35) collected between 5 May 2020, and 31 January 2022 were analyzed. Genomic sequences were assigned to phylogenetic clades using NextClade and to Pango lineages using pangolin. Phylogenetic and phylogeographic analyses were performed to determine the geographical sources and time of virus introduction in Burkina Faso. Results: The analyzed SARS-CoV-2 genomes can be assigned to 10 phylogenetic clades and 27 Pango lineages already described worldwide. Our analyses revealed the important role of cross-border human mobility in the successive SARS-CoV-2 introductions in Burkina Faso from neighboring countries. Conclusions: This study provides additional insights into the genomic epidemiology of SARS-CoV-2 in West Africa. It highlights the importance of land travel in the spread of the virus and the need to rapidly implement preventive policies. Regional cross-border collaborations and the adherence of the general population to government policies are key to prevent new epidemic waves.

## 1. Introduction

The Severe Acute Respiratory Syndrome coronavirus 2 (SARS-CoV-2) is the etiological agent of coronavirus disease 2019 (COVID-19) [1]. Following its initial detection in Wuhan, China, in December 2019 this novel virus has rapidly spread and continues to cause successive epidemic waves worldwide [2]. The first COVID-19 case in Africa was recorded in Egypt on 14 February 2020, followed by case reports from many sub-Saharan countries in the following few days [3,4].

Burkina Faso is a landlocked country in West Africa, and its capital and largest city is Ouagadougou. The first COVID-19 case (a traveler returning from France) was recorded in Ouagadougou on 9 March 2020. The first death was confirmed on 18 March 2020 [5]. Eleven days later, the first COVID-19 case in western Burkina Faso was recorded in Bobo Dioulasso (the largest city in western Burkina Faso) and concerned a traveler from Ouagadougou. On 21 March 2020, Burkina Faso officials declared a countrywide curfew (from 7 p.m. to 5 a.m.). Stringent control measures were introduced in the following days, including a public gathering ban and quarantine in cities with at least one reported COVID-19 case. Border closure and a ban on international passenger flights were imposed on 15 April 2020 [6]. These measures effectively curbed the epidemic at its earliest stages, and COVID-19 incidence remained low in the following months. From 13 May 2020, restrictions were partially lifted. Restaurants and public places were reopened concomitantly with the introduction of social distancing measures and the use of personal protection equipment. On 1 August 2020, international passenger flights were resumed with containment, self-containment, or isolation measures, if required [7]. Despite these sanitary measures, incidence began to increase sharply and the second “wave” of the epidemic hit the country from September to October 2020. This episode was followed one month later by a third and more violent wave after which incidence declined first rapidly from mid-January to mid-February 2021 and then more slowly before falling to basal rates from May to August 2021. The number of new cases increased again during the last trimester of 2021 when the fourth (September–November 2021) wave hit the country. The fifth wave started few days after border reopening on 1 December 2021 [8], reached its peak on 9 January 2022, and ended in March 2022 [9].

From the beginning of the epidemic, Burkina Faso has deployed substantial efforts in local capacity building in order to deal with this novel virus. Along with epidemiological analysis based on case reports, implementing genomic sequencing was considered as a priority to understand the transmission dynamics of the virus and ensure effective surveillance. Both North–South and South–South collaborations were carried out to achieve this objective. In the scope of the African Network for improved Diagnostics, Epidemiology and Management of Common Infectious Agents (ANDEMIA, Bouake, Cote d’Ivoire), collaborators from the Robert Koch institute, Berlin, Germany trained between November 2020 and March 2021 laboratory personnel and scientists from Centre Muraz (CM), Bobo Dioulasso, Burkina Faso and Souro Sanou University Hospital Centre in Bobo Dioulasso (SSUHC), Bobo Dioulasso, Burkina Faso on SARS-CoV-2 whole-genome sequencing using the ARTIC protocol and a MinION M1kC device (Oxford Nanopore Technologies, Oxford, UK). On 26 November 2020, the first SARS-CoV-2 genomic sequences from Burkina Faso were submitted to the global initiative on sharing avian influenza data (GISAID) database [10].

In this study, we provide a genomic epidemiology study of SARS-CoV-2 from the region of western Burkina Faso by analyzing 372 viral genomes obtained from isolates collected between 5 May 2020 and 31 January 2022. We carried out phylogenetic analyses to infer the geographic sources and timing of virus introductions in Burkina Faso.

## 2. Materials and Methods

### 2.1. Study Area

The SSUHC and CM are both located in the city of Bobo Dioulasso, capital of the Hauts-Bassins region and the economic capital of Burkina Faso. The health coverage regions of the SSUHC, which includes the Haut-Bassins, the Cascades, Boucle du Mouhoun, and Southwest regions with a total population estimated at 6,555,016 inhabitants in 2022. This center, with a capacity of 656 beds, has six departments namely medicine, pediatrics, surgery, obstetrics and reproductive medicine, pharmacy, and laboratory. In the context of the coronavirus pandemic, the virology unit, part of the laboratory department in addition to routine diagnosis, was the coordinating center for the diagnosis and surveillance of SARS-CoV-2 variants in the west and south of the country.

### 2.2. Ethical Approval

This study was carried out by collecting data from patient records as part of the COVID-19 surveillance. Patients data was anonymized before analysis an only the laboratory number was mentioned to ensure confidentiality. The results were shared with the Ministry of Health, Public Hygiene, and Welfare as part of the routine surveillance of SARS-CoV-2 variants. Data collection was performed in the frame of studies conducted by ANDEMIA, which adheres to the tenets of the Declaration of Helsinki, as well as any national legislation and ethical standards. The study protocol was therefore approved by the Burkina Faso national ethics committee (Comité d’Ethique pour la Recherche en Santé (2017–5-057)).

### 2.3. Sampling and SARS-CoV-2 Screening

COVID-19 testing was carried out on incoming travelers, on suspected cases according to WHO definition [11] and on individuals that were listed as close contact of a confirmed case. Testing was also made available on a voluntary basis for non-suspect individuals. The socio-demographic information collected included age, gender, place of residence, and status (suspect case and non-suspect case according to WHO). Nucleic acids were extracted from nasopharyngeal swab samples and were tested using real-time Polymerase Chain Reaction (PCR) for SARS-CoV-2. Different PCR kits were used depending on their availability on the market: (i) Detection Kit for 2019 Novel Coronavirus (2019-nCoV) RNA (PCR-Fluorescence Probing) Daan Gene Co., Ltd., Sun Yat-Sen University, Guangzhou, China; (ii) Abbott RealTime SARS-CoV-2 Assay, Abbott Molecular Inc., Des Plaines, IL, USA; (iii) Real-Time Fluorescent RT-PCR Kit for Detecting SARS-CoV-2, Beijing Genomics Institute (BGI), Beijing, China; (iv) Liferiver Novel Coronavirus (2019-nCoV) Real Time Multiplex RT-PCR Kit, ZJ Bio-Tech Co., Ltd., Shanghai, China; (v) Novel Coronavirus (2019-nCoV) Nucleic Acid Diagnostic Kit (PCR-Fluorescence Probing) Sansure Biotech. Inc., Changsha, China; (vi) TIB-Molbiol SARS-CoV-2 RT-PCR Kit TIB Molbiol, Berlin, Germany; and (vii) FastPlex Triplex SARS-CoV-2 Detection Kit (RT-Digital PCR), PreciGenome LLC, San Jose, CA, USA.

### 2.4. Sequencing and Genome Assembly

Samples with real-time PCR cycle threshold (Ct) score below 35 were selected for sequencing. Sequencing was carried out by the SSUHC and the CM using the ARTIC protocol and Oxford Nanopore Technology MinIon MK1B and MK1C. The ARTIC Network bioinformatic pipeline [12] was used for genome assembly and variant calling steps. Genomes with a minimum of 50% of coverage were submitted to GISAID.

### 2.5. Phylogenetic and Phylogeographic Analyses

Genomic sequences were assigned to phylogenetic clades using NextClade (version 0.13.0) and to Pango lineages using the pangolin toolkit (version 2.3.2) with pangoLEARN (version v10 February 2021). 

To infer the geographic sources of SARS-CoV-2 introduction in western Burkina Faso, the SARS-CoV-2 genomic sequences from worldwide that were closest to those from our study were introduced in the analysis. As the number of available SARS-CoV-2 genomic sequences is exceptionally high (8,209,303 complete genomic sequences on GISAID on 9 March 2022), a computational rationale approach was followed to subsample the complete global dataset available on GISAID. Specifically, curated Nextstrain continent-representative datasets were downloaded from the GISAID database (9 March 2022) for all six continents (2620 to 3937 genomes per continent). Moreover, all the global sequences of Pango lineages identified in western Burkina Faso SARS-CoV-2 genomes were included after carrying out a lineage-based search of genomic sequences in the GISAID database. Only complete SARS-CoV-2 genomes (defined by a length > 29,500 nucleotides) and those with complete information on the collection date were downloaded. Burkina Faso sequences from our study and the downloaded global sequences were aligned against the reference Wuhan Hu-1 genome using the uvaialign function of the uvaia software v1 (Quadram Institute Bioscience, Norfolk, UK). Uvaia was also used to identify the closest sequences to the western Burkina Faso sequences in the global SARS-CoV-2 dataset; at most five global sequences for each Burkina Faso sequence were selected. When two or more global sequences from the same country were identical, only one was included in the analysis.

A Maximum Likelihood (ML) phylogenetic tree was generated for the dataset using IQTREE2 and a General Time-Reversible (GTR) nucleotide substitution model that took into account the inter-site heterogeneity through a discretized gamma distribution with four rate categories (GTR + Γ). This model was identified as the best fitting model for ML inference by jModelTest v2.1.10. Branch support was inferred using 1000 bootstrap replicates. The ML tree was analyzed with TempEst to identify the temporal signals of a dataset and possible outlier sequences (e.g., sample contamination, data annotation errors, sequencing and alignment errors, or assembling issues).

The GTR + Γ substitution model was then used to derive a dated SARS-CoV-2 phylogeny using the Bayesian Markov Chain Monte Carlo (MCMC) approach implemented in BEAST v1.10.4. An uncorrelated lognormal relaxed clock was used with a coalescent tree prior under constant growth. Five replicate runs were performed for each 100 million MCMC steps with sampling parameters and trees every 10,000 steps. The convergence of all parameters was assessed by calculating the effective sample sizes. Their threshold was set at 200 after discarding the initial 10% of each run as a burn-in using Tracer v1.7.1.

The phylogeography was reconstructed from the time-scaled tree generated using PastML, a fast ancestral reconstruction tool that implements ML-based ancestral reconstruction (ACR) methods. ACR was carried out using the maximum likelihood marginal posterior probabilities approximation (MPPA) method with an F81-like model (https://pastml.pasteur.fr/) (accessed on 17 May 2022). To reconstruct the ancestral character states and their changes along the trees, each taxon was assigned to its sampling location character, and infections detected in incoming travelers were considered to have occurred in the country of departure.

## 3. Results

### 3.1. SARS-CoV-2 Genetic Diversity in Western Burkina Faso

In total, 2242 PCR-positive nasopharyngeal samples were recorded in western Burkina Faso between 12 May 2020 and 19 January 2022, most of them were from Bobo Dioulasso (*n* = 1797), the largest city in the region. The mortality rate during this period was 3.4% (76/2242; 95% confidence interval [CI]: 2.6–4.1). A total of 503 samples were sequenced, from which 372 passed the GISAID quality control (minimum of 50% genomic coverage). Out of them, 173 (46.5%) were from female participants and 199 (53.5%) from male participants with similar mean ages, 41.99 (7–93) and 42.10 (8–88) years of age, respectively. Most of these SARS-CoV-2 sequences were obtained from samples collected in Bobo Dioulasso (*n* = 343) and the others from eight cities in western Burkina Faso (*n* = 21) and from samples collected at land borders from travelers returning from Côte d’Ivoire (*n* = 7) and at Bobo Dioulasso airport from a traveler coming from France (*n* = 1).

**Figure 1:**
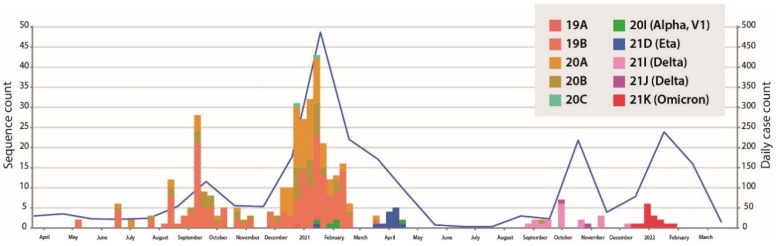
COVID-19 confirmed cases and SARS-CoV-2 genetic diversity in western Burkina Faso. Timeline showing the collection dates of the confirmed COVID-19 cases in western Burkina Faso from 9 March 2020 to 20 March 2022 and the during this period (blue line). The colors of bars represent the clades (NextClade nomenclature) that were identified and the heights of bars correspond to the number of sequenced samples each week and passing the global initiative on sharing avian influenza data (GISAID) quality control.

The 372 genomes were assigned to 10 different phylogenetic clades (Figure 1) using the NextClade classification system that included 30 clades described worldwide on 20 October 2022 [13]. Although nearly 50% of sequences (*n* = 184) had a relatively high proportion of missing data (>3000 Ns), clade signature mutations were in sufficient numbers to ensure good phylogenetic support for the NextClade classification (Figure S1a). Clade 19B was the first clade to be detected in western Burkina Faso (8 May 2020). This clade was prevalent in the region for almost one year (until 18 March 2021) and was the most common clade among samples collected during the first three epidemic waves. During the same period, clades 20A and 20B also were identified, although they were less common. Then, the number of new cases dropped sharply from mid-January to mid-February 2021, and then more slowly from mid-February to mid-May 2021. This slowdown coincided with the rise of clade 21D (Eta) from mid-March to mid-April 2021. Samples collected during the fourth wave revealed that the majority of circulating SARS-CoV-2 viruses belonged to clade 21I (Delta), followed by clade 21J (Delta). SARS-CoV-2 viruses from the fifth wave belonged mainly to clade 21K (Omicron).

A total of 313 SARS-CoV-2 genomes could be classified in 27 Pango lineages whereas 59 remained unclassified. A Pango lineage is defined by the carriage of specific SNPs, the introduction and circulation in a new region, and the constitution of a well-supported monophyletic group. Many genomes clustered together, although they were classified as distinct Pango lineages (Figure S1b). The GISAID quality standards consider that 50% of confident calls is sufficient and is the minimum amount of data to be phylogenetically useful. However, this threshold probably allows the inclusion of many genomes without enough specific SNPs for accurate Pango classification, leading to a high proportion of misclassified genomes. According to the authors at the origin of the Pango nomenclature, sequences with more than 30% of missing data should not be used for lineage classification [14]. Yet, many mismatches between lineage classification (Pango) and phylogeny persisted even after using this threshold (data not shown). Therefore, a conservative subsampling of our dataset was carried out by only including genomes with more than 90% of confident calls for all subsequent analyses. This curated dataset included 188 genomes belonging to 19 Pango lineages and one unclassified genome. This curation step resulted in a significant improvement of the phylogenetic support of Pango lineage classification (Figure S1c). Five of these lineages were mainly regional lineages, previously described in different West African countries and that were identified in western Burkina Faso between mid-2020 and early 2021 Figure S2: (i) A.18 that included 16 SARS-CoV-2 genomes in GISAID among which 14 were from Côte d’Ivoire; (ii) A.19 that included 62 SARS-CoV-2 genomes in GISAID, among which 49 were from Côte d’Ivoire; (iii) B.1.1.359 that included SARS-CoV-2 33 genomes in GISAID, among which 24 were from Ghana; (iv) B.1.388 that included 9 SARS-CoV-2 genomes in GISAID, among which seven were from Côte d’Ivoire; and (v) B.1.416 that included 686 SARS-CoV-2 genomes in GISAID among which 183 were from Senegal and 162 from Gambia. B.1.1.7 lineage (Alpha) carrying spike mutations N501Y, D614G, and P681H was also identified in the region. This variant was considered by the WHO as a variant of concern (VOC) between 18 December 2020 and 9 March 2022 since it was associated with increased transmissibility and disease severity in other countries [15,16,17]. In western Burkina Faso, this VOC was not apparently involved in important local transmission since only a single genome of B.1.1.7 lineage was identified in the region (three genomes if we include those having between 10% and 50% of Ns).

### 3.2. Geographic Sources of Virus Introduction into Western Burkina Faso

The 188 high-quality genomic sequences collected in western Burkina Faso were then analyzed in the context of the global SARS-CoV-2 genetic diversity. By using our subsampling approach, 237 global sequences available in GISAID were selected according to quality and genetic closeness criteria. The regression of root-to-tip genetic distance against sampling dates revealed two outlier sequences that were excluded from the dataset. The curated dataset (*n* = 423) included 187 sequences obtained in western Burkina Faso (this study) and 236 global sequences and exhibited a strong temporal signal (r^2^ = 0.88) (Figure S3).

The ML phylogeographic analyses estimated the occurrence of at least 44 independent SARS-CoV-2 introductions into western Burkina Faso (Figure S4). Twenty-six SARS-CoV-2 genomes from western Burkina Faso did not cluster with any other genome from the same region. These viruses can represent introductions not involved in forward transmission within the region or with undetectable transmission due to low sampling. Therefore, they were designed as singletons. Besides singletons, 18 independent introductions constituted clusters of two or more genomes, probably indicating lineages involved in local transmission (Table 1). For five of these clusters that included 65 sequences from western Burkina Faso in total, a regional origin was inferred: from Cote d’Ivoire (*n* = 4) and from Niger (*n* = 1). For five clusters that included 36 sequences from western Burkina Faso, a European origin was inferred. The remaining eight clusters, which comprised 53 sequences from western Burkina Faso, could not be associated with a specific geographic origin.

**Figure 2:**
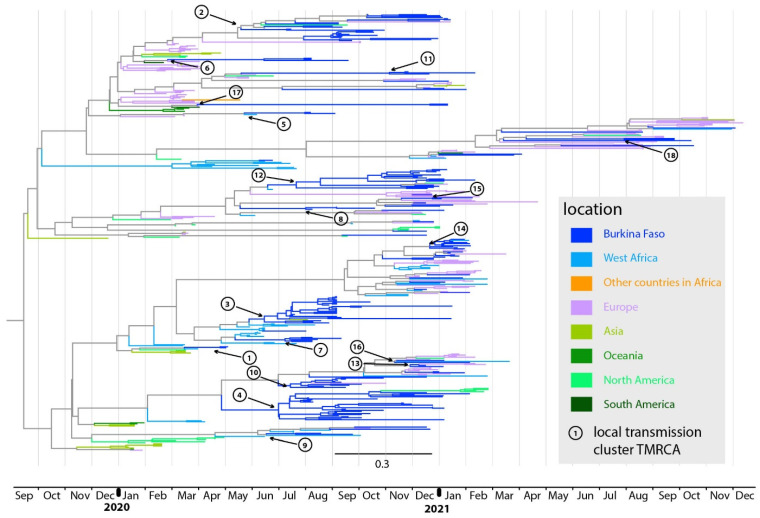
Dated phylogenetic tree showing the 18 transmission clusters identified in western Burkina Faso. The tree branches are color-coded according to each geographic region. The estimated time to the most recent common ancestor (TMRCA) values of Burkina Faso transmission clusters are highlighted by black arrows and circles with consecutive numbering according to the detection date (sample with the earliest collection date within that cluster; see Table 1).

The dated phylogenetic tree (Figure 2) was visually inspected to retrieve these 18 clusters inferred from the ML phylogeographic analyses. Their detection lags (time elapsed between the inferred transmission cluster time, the time to the most recent common ancestor, TMRCA, and the collection date of its earliest sampled sequence) ranged between 3 and 265 days (Table 1), with a median of 73 days (IQR: 18–141 days). Sampling limitations and the low genetic divergence observed in SARS-CoV-2 over short time spans [18,19,20] could have blurred the distinction between clusters resulting from a single initial independent introduction and clusters constituted from multiple introductions of closely related viruses. This could have led to an underestimation of the number of independent introductions. The dated phylogenetic tree showed that ten clusters inferred from ML phylogeographic analyses were only composed of Burkina Faso sequences, suggesting a strictly local transmission of these clusters. Conversely, eight clusters included sequences from international locations and western Burkina Faso, suggesting multiple independent introductions of genetically similar viruses from shared sources.

### 3.3. Timing of Virus Introductions into Western Burkina Faso

Inferring the precise importation date of a virus is challenging, especially when the sample size is limited. The importation date of a virus at the origin of a local transmission cluster falls between the preceding ancestral node occurring outside the country (lower bound) and the most recent common ancestor (MRCA) node of Burkina Faso sequences that compose the cluster (upper bound). For the four introductions that originated from neighboring Côte d’Ivoire and that developed infection clusters in Burkina Faso (Table 1), the two bounds delimiting the likely introduction time of seeding viruses felt within the period of ban on international passenger flights (23 March to 1 August 2020). In three of these clusters, sequences obtained from returning travelers at land borders with Côte d’Ivoire were positioned basally to Burkina Faso sequences in the dated phylogenetic tree. This finding is consistent with the hypothesis of a role of cross-border mobility in SARS-CoV-2 introductions into Burkina Faso. For the single introduction that originated from neighboring Niger, the two bounds delimiting the likely introduction time of the virus at the origin of a local cluster in Burkina Faso felt between the date of airport reopening on 1 August 2020 and the date of land border reopening on 1 December 2021. During this period, both air travelers and illegal land travelers from these countries could have been at the origin of viral introductions, without possible distinction between these routes. Introductions from other locations could not be established for this period because none or only one of the two introduction time bounds fell within the flight ban period (Table 1).

### 3.4. Geographic Distribution of Transmission Lineages in Western Burkina Faso

Most high-quality Burkina Faso sequences included in this study were from Bobo Dioulasso (*n* = 159; 85.0%) and only 21 (11.2%) were from one of eight other cities in the region of western Burkina Faso. Among the 26 singletons identified in this study, 25 were from Bobo Dioulasso. Among the 18 clusters inferred from the ML phylogeographic analyses, 10 included sequences only from Bobo Dioulasso, six comprised sequences from Bobo Dioulasso and another city, and two included sequences from Bobo Dioulasso and two or more other cities (Table 1). This pattern suggests an effective inter-province viral circulation/transmission in which Bobo Dioulasso is the central hub.

## 4. Discussion

Since the publication of the first SARS-CoV-2 genome, scientists around the world have quickly understood the crucial need to monitor the evolution of this rapidly spreading novel virus. In western Burkina Faso, viral genome sequencing was conducted between 12 May 2020 and 31 January 2022 in the framework of the SARS-CoV-2 surveillance efforts carried out in this region. In this retrospective study, we took advantage of these sequencing data to describe patterns of SARS-CoV-2 introduction and spread in this region, and to evaluate the efficiency of COVID-19 control policies implemented in the country.

The aim of the first COVID-19 control policies decided by the Burkina Faso government was to prevent new SARS-CoV-2 introductions from abroad and to disrupt transmission chains inside the country. However, by the time such measures (border closure and ban on air travel) were implemented (23 March 2020), our results reveal that several independent introductions had already occurred from international locations. These early introductions were predominantly from Europe, as observed in most sub-Saharan African countries during the initial phases of the epidemic [21]. The absence of new introductions from remote international locations during the period of airport closure suggests that the air travel ban was efficient. Border closure was less successful because several introductions were associated with cross-border land travels from neighboring Côte d’Ivoire. Two Pango lineages predominantly found in Côte d’Ivoire (A.18 and A.19) were found involved in several introduction events. Due to its geographical position, Burkina Faso is a crossroad between coastal countries and landlocked countries and the connectivity provided by land travel is a major means of human mobility. Noteworthy, most incoming (regular and irregular) travelers in Burkina Faso come from Côte d’Ivoire because the two countries share a common border that stretches across 594 km [22]. The persistence of illegal land travel despite border closure and its major role in SARS-CoV-2 introductions in Burkina Faso have been an important concern, such as in other African countries [23,24,25]. It highlights the challenge of obtaining the general population’s adherence to some governmental policies, which is paramount for their efficacy.

In Burkina Faso, most SARS-CoV-2 genomic sequences were obtained from samples collected in Bobo Dioulasso and very few sequences (or none) were available from many neighboring provinces. This can be explained by the fact that the city of Bobo Dioulasso hosts the SSUHC, a reference center for COVID-19 diagnosis. All these unrepresented or underrepresented locations could have been the point of entry for new introductions, at the origin of new clusters. Moreover, in most West African countries, sampling was very limited throughout the epidemic period (e.g., 635 and 156 complete genomic sequences from Côte d’Ivoire and Niger, respectively, on GISAID on 9 March 2022). These sampling limitations could have restricted our capacity to infer the geographic sources of virus introductions in several cases [26]. Additionally, screening was not systematic, resulting in no or very few samples sequenced during certain periods or for certain geographic locations. This sampling bias might have resulted in a delayed identification of some clusters. This is illustrated by the large variations in detection lags, indicating that some transmission clusters were not detected immediately after their emergence in the country.

Such as in many other African countries, whole genome sequencing of positive SARS-CoV-2 samples was carried out using ONT, due to its suitability for rapid deployment in resource-limited settings [27]. ONT devices are relatively low-cost, portable, do not require specific laboratory infrastructure or advanced technical skills [28], and have already proven their relevance in previous epidemic situations (e.g., Ebola, Zika) [29,30]. The main concern regarding this technology is its base calling accuracy, due to conflicting calls at the same position that lead to high proportions of uncalled positions (Ns). This was observed in a substantial number of genomes included in this study (see results), impacting for example the accuracy of Pango lineage assignment. This issue could have led to the missed detection of new variants of concern especially in the context of low sequencing rates as it is the case in western Burkina Faso, which can substantially undermine the surveillance efforts. The Ns can also be explained by primer dropout due to the fast-evolving nature of SARS-CoV-2 [31]. Increasing sequencing depth would decrease the proportion of uncalled positions (Ns). For instance, it was shown that reaching a minimum of ~60-fold coverage depth efficiently solves this issue and allows highly accurate consensus-level sequence determination [32].

This study provides data on SARS-CoV-2 genomic diversity in Burkina Faso and shows the substantial diversity of circulating lineages, all being also found in other countries of the world at the time of study. Our results suggest that neighboring countries played a major role in SARS-CoV-2 introductions into Burkina Faso. Cross-border land travels have at least partially contributed to these introductions that were probably at the origin of several local transmission clusters in western Burkina Faso. Regional collaborations and the general population’s adherence to governmental policies may be required to prevent additional SARS-CoV-2 introductions into Burkina Faso.

## Figures and Tables

**Table 1:** Transmission clusters identified in western Burkina Faso.

Transmission Cluster	Pango Lineage	Geographic Origin	TMRCA with the Most Likely Source Populations from Outside of the Country	Cluster Median TMRCA	Earliest Collection Date	Last Collection Date	Detection Lag (in Days)	Town
1 (*n* = 2)	A	Unknown	ND	1 May 2020	8 May 2020	10 May 2020	ND	Bobo Dioulasso
2 (*n* = 26)	B.1.1.404	England	8 March 2020	20 May 2020	21 July 2020	22 January 2021	73	Bobo Dioulasso; Boromo; Gaoua; Houndé
3 (*n* = 25)	A.19	Cote d’Ivoire	2 June 2020	20 June 2020	23 July 2020	20 January 2021	18	Bobo Dioulasso; N’Dorola; Orodara; Diebougou
4 (*n* = 27)	A.21	Unknown	ND	6 July 2020	10 August 2020	9 February 2021	ND	Bobo Dioulasso; Orodara
5 (*n* = 2)	B.1.388	Unknown	ND	5 August 2020	12 August 2020	9 September 2020	ND	Bobo Dioulasso; Houndé
6 (*n* = 3)	B.1.1	England	8 March 2020	11 July 2020	13 August 2020	24 September 2020	125	Bobo Dioulasso; Houndé
7 (*n* = 5)	A.19	Cote d’Ivoire	1 May 2020	13 July 2020	14 August 2020	16 September 2020	73	Bobo Dioulasso
8 (*n* = 6)	B.1	Unknown	ND	1 August 2020	14 August 2020	6 January 2021	ND	Bobo Dioulasso; Banfora
9 (*n* = 2)	A.18	Cote d’Ivoire	22 July 2020	25 July 2020	24 August 2020	29 September 2020	3	Bobo Dioulasso
10 (*n* = 10)	A.21	Unknown	ND	5 July 2020	27 August 2020	22 December 2020	ND	Bobo Dioulasso
11 (*n* = 3)	B.1.1	England	24 May 2020	9 November 2020	1 December 2020	15 February 2021	169	Bobo Dioulasso
12 (*n* = 17)	B.1	Cote d’Ivoire	23 June 2020	26 July 2020	7 December 2020	15 February 2021	33	Bobo Dioulasso; Boromo
13 (*n* = 2)	A.21	Unknown	ND	2 December 2020	21 December 2020	10 January 2021	ND	Bobo Dioulasso
14 (*n* = 16)	A.27	Niger	22 October 2020	1 November 2020	30 December 2020	10 February 2021	10	Bobo Dioulasso
15 (*n* = 2)	B.1	Unknown	ND	28 December 2020	8 January 2021	12 February 2021	ND	Bobo Dioulasso; Batié
16 (*n* = 2)	A.21	Unknown	ND	15 November 2020	11 January 2021	9 February 2021	ND	Bobo Dioulasso
17 (*n* = 2)	B.1.1.359	Scotland	4 April 2020	25 December 2020	15 January 2021	15 January 2021	265	Bobo Dioulasso
18 (*n* = 2)	AY.133	Denmark	14 March 2021	2 August 2021	29 september 2021	29 September 2021	141	Bobo Dioulasso

ND: not determined.

## Data Availability

SARS-CoV-2 genomes analyzed in this study are available on the global initiative on sharing avian influenza data (GISAID) database (https://gisaid.org/).

## Supplementary Materials

The following supporting information can be downloaded at: https://www.mdpi.com/article/10.3390/v14122788/s1, Figure S1: Phylogenetic relationship amongst the 372 SARS-CoV-2 samples collected in western Burkina Faso. Maximum-likelihood tree Branch support was inferred using 1000 bootstrap replicates. (a) Branch tips are colored according to their clade (NextClade nomenclature). (b) Branch tips of all SARS-CoV-2 genome sequences colored according to their Pango lineage. (c) Branch tips only of sequences covered by more than 90% of confident calls (n = 188) colored according to their Pango lineage; Figure S2: Pango lineages timeline of SARS-CoV-2 genomes collected in western Burkina Faso. The colors of bars represent the Pango lineages that were identified and the heights of bars correspond to the number of sequenced samples each week. Only genomes passing the global initiative on sharing avian influenza data (GISAID) quality control and covered by more than 90% of confident calls are included (n = 188); Figure S3: Root-to-tip genetic distances (based on a heuristically rooted maximum likelihood tree) versus sample collection dates for the SARS-CoV-2 genome sequences used for the phylogenetic and phylogeographic analyses. Red, sequences of samples collected in western Burkina Faso; gray, sequences from the entire world; black line, linear regression trendline; Figure S4: Ancestral reconstruction and estimation of the number of SARS-CoV-2 introductions into Burkina Faso. Estimated introductions based on the ancestral reconstruction using PastML (MPPA + F81) with geolocations aggregated by country (a color for each country). Internal nodes are collapsed based on the shared reconstructed ancestry and the edges denote putative transmission events. The figure at the top (initial output) has been divided into five parts (a–e), each enlarged and shown separately for better visualization.
