# Supplementary material for: Genomic Epidemiology of SARS-CoV-2 in Western Burkina Faso, West Africa
Source: Viruses. 2022 Dec 14;14(12):2788. doi: 10.3390/v14122788 (PMC9782145; doi:10.3390/v14122788)
Supplement: Supplementary file 1 [file viruses-14-02788-s001.zip › Figure-S4b.pdf]

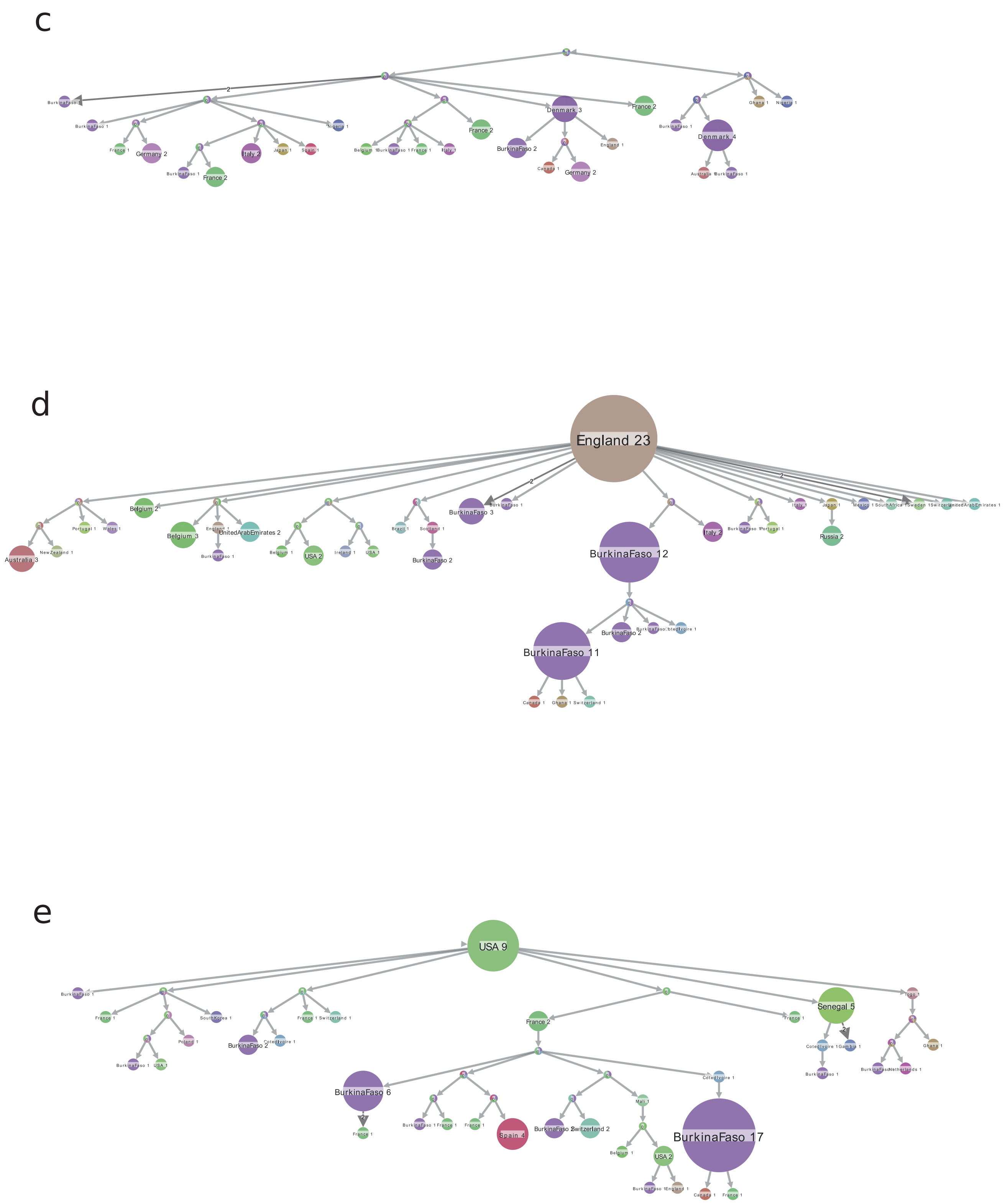
c
Denmark 3
Denmark 4
France 2
Germany 2
Italy 2
BurkinaFaso 2
d
England 23
BurkinaFaso 12
BurkinaFaso 11
BurkinaFaso 3
BurkinaFaso 2
Belgium 2
Belgium 3
UnitedArabEmirates 2
USA 2
Australia 3
Italy 2
Russia 2
e
USA 9
Senegal 5
France 2
BurkinaFaso 6
BurkinaFaso 17
BurkinaFaso 2
Spain 4
Switzerland 2
USA 2
