# Supplementary figures and images for: Genomic Epidemiology of SARS-CoV-2 in Western Burkina Faso, West Africa
Source: Viruses. 2022 Dec 14;14(12):2788. doi: 10.3390/v14122788 (PMC9782145; doi:10.3390/v14122788)

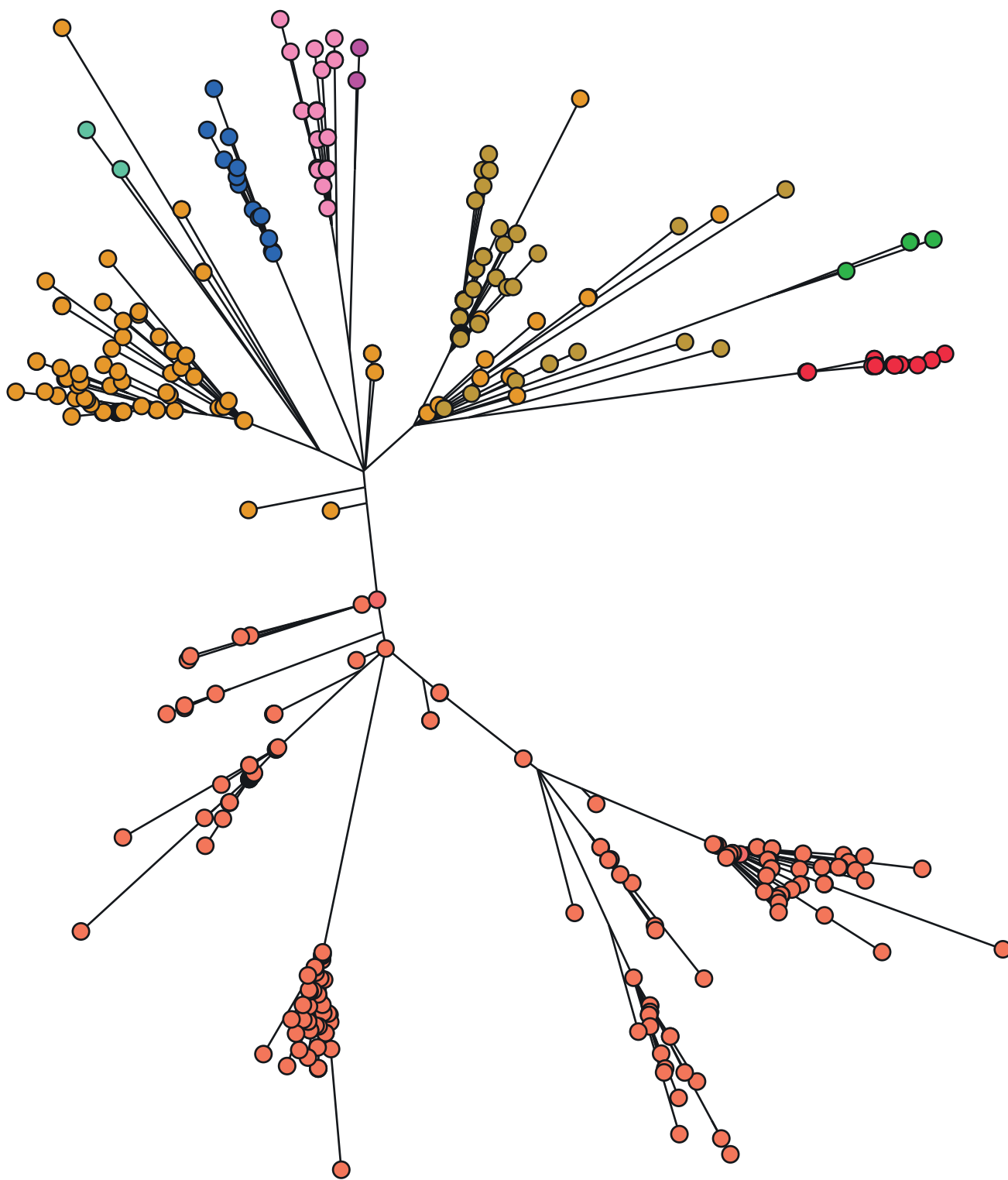

- |     |                 |
|-----|-----------------|
| 19A | 20I (Alpha, V1) |
| 19B | 21D (Eta)       |
| 20A | 21I (Delta)     |
| 20B | 21J (Delta)     |
| 20C | 21K (Omicron)   |

Supplement: Supplementary file 1 [file viruses-14-02788-s001.zip › Figure-S1a.pdf]

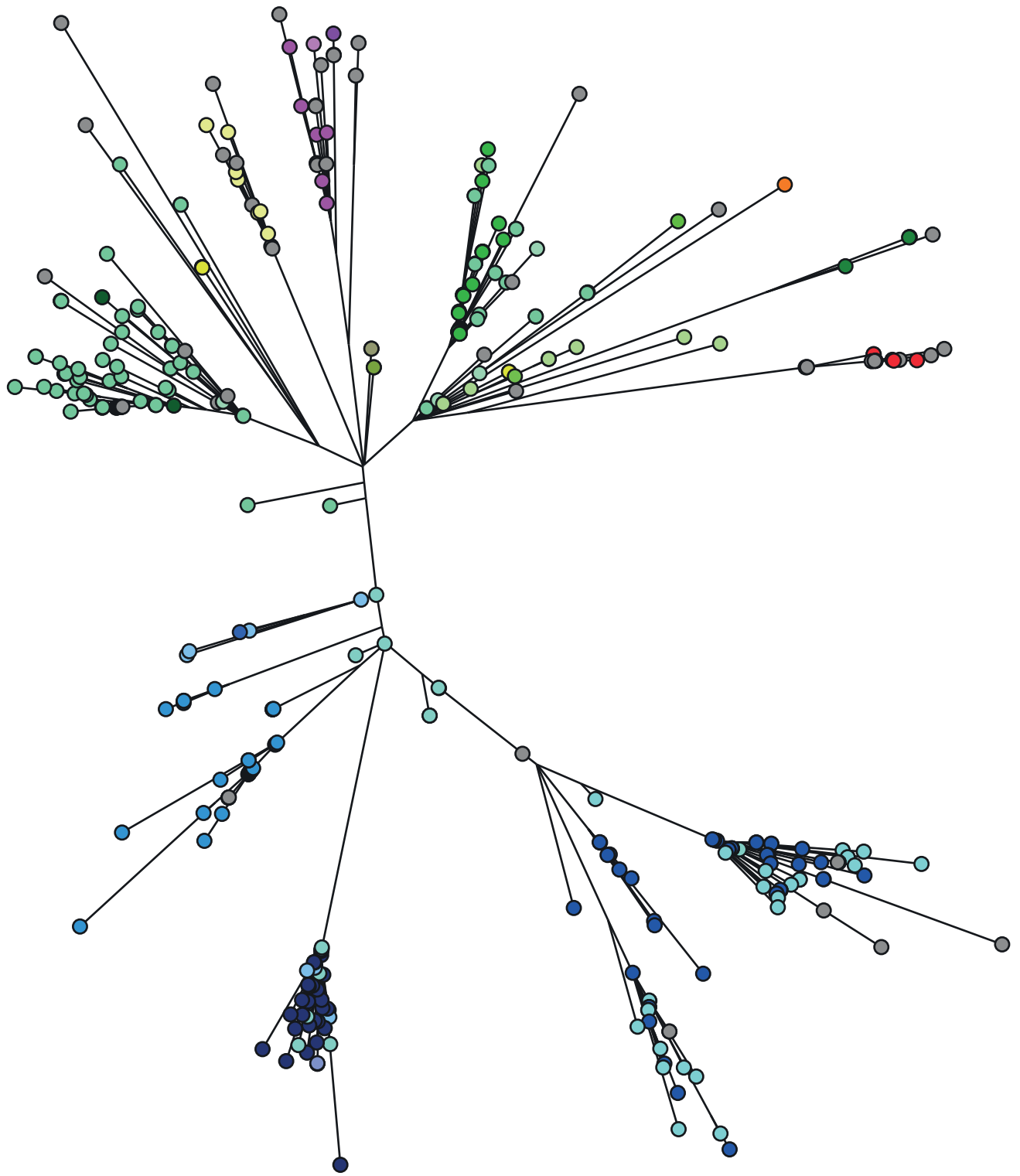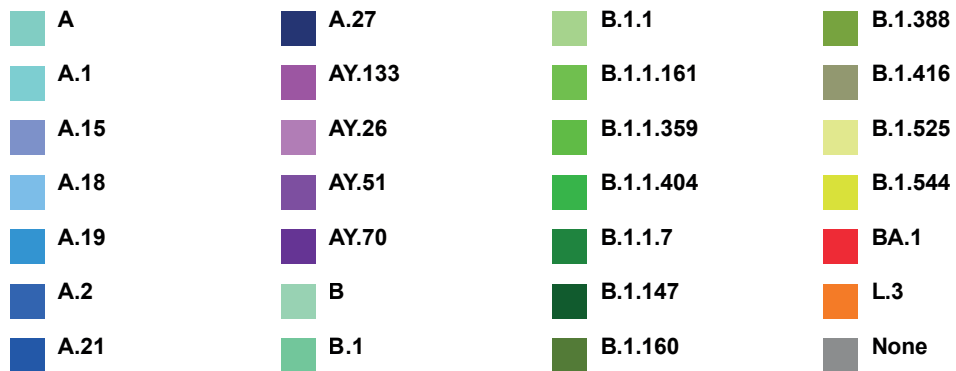

Supplement: Supplementary file 1 [file viruses-14-02788-s001.zip › Figure-S1b.pdf]

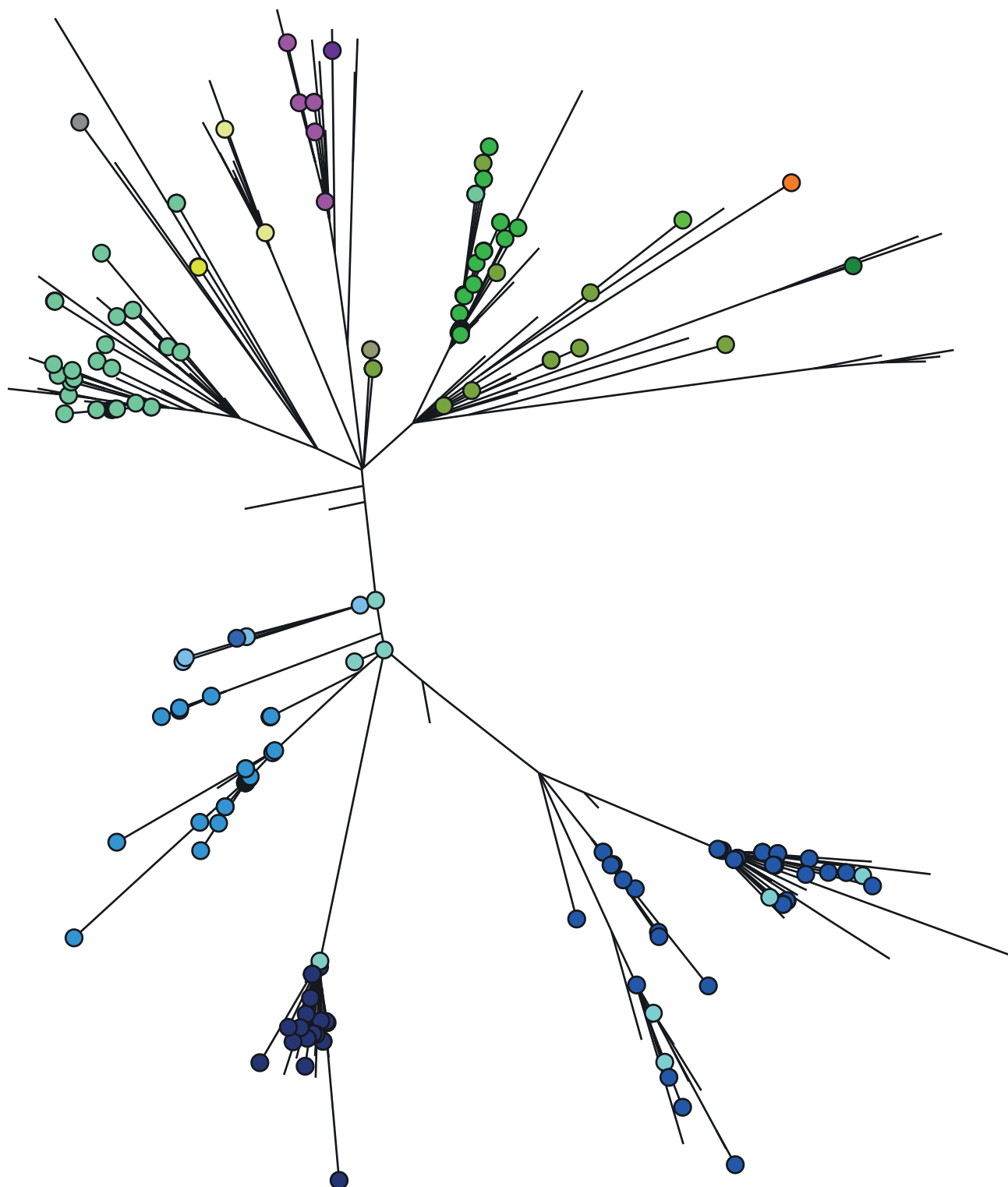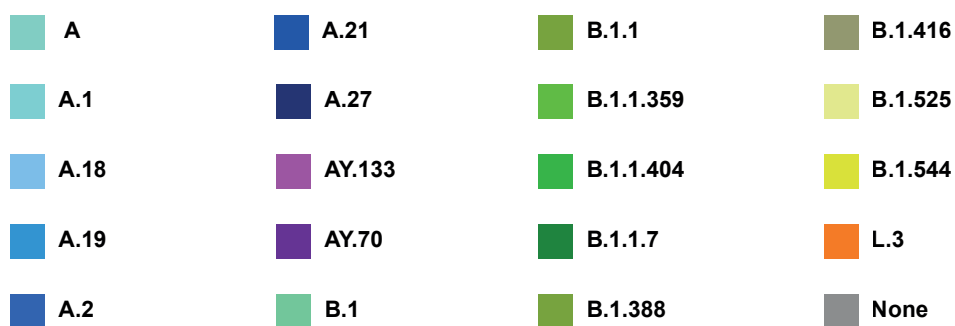

Supplement: Supplementary file 1 [file viruses-14-02788-s001.zip › Figure-S1c.pdf]

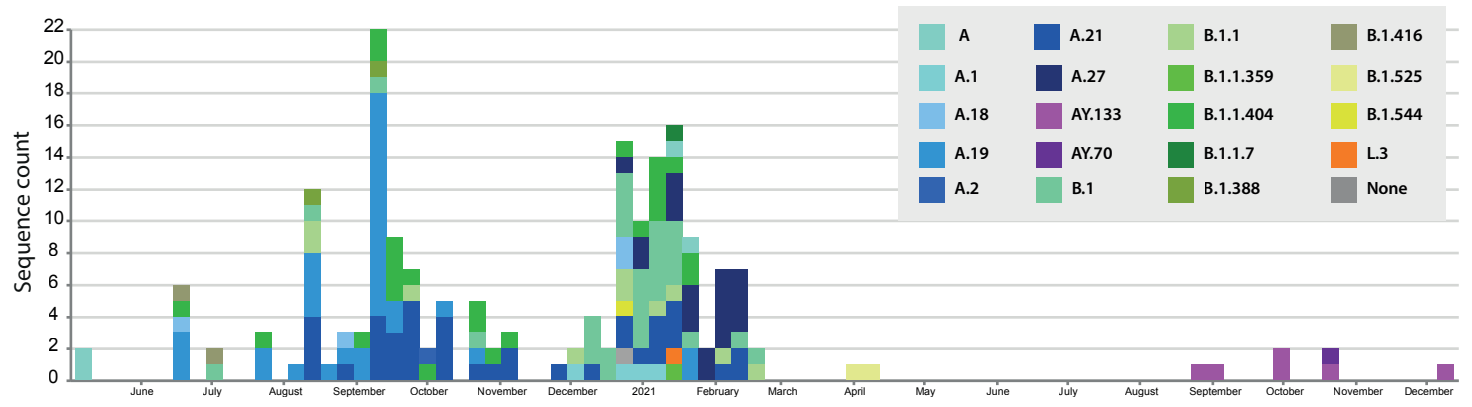

Supplement: Supplementary file 1 [file viruses-14-02788-s001.zip › Figure-S2.pdf]

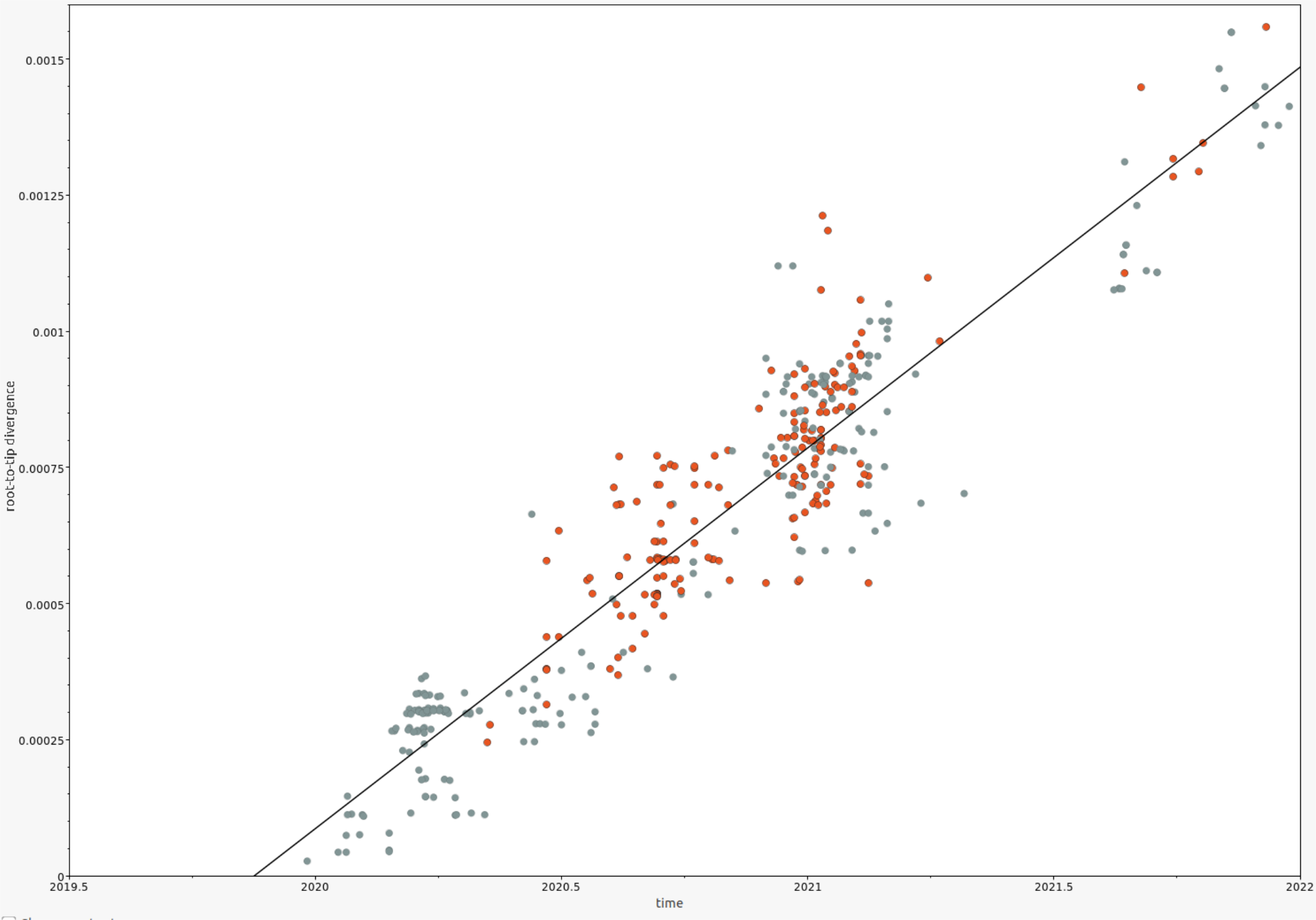

Supplement: Supplementary file 1 [file viruses-14-02788-s001.zip › Figure-S3.png]
