# Supplementary material for: Genomic Epidemiology of SARS-CoV-2 in Western Burkina Faso, West Africa
Source: Viruses. 2022 Dec 14;14(12):2788. doi: 10.3390/v14122788 (PMC9782145; doi:10.3390/v14122788)
Supplement: Supplementary file 1 [file Figure-S4a.pdf › Figure-S4a.pdf]

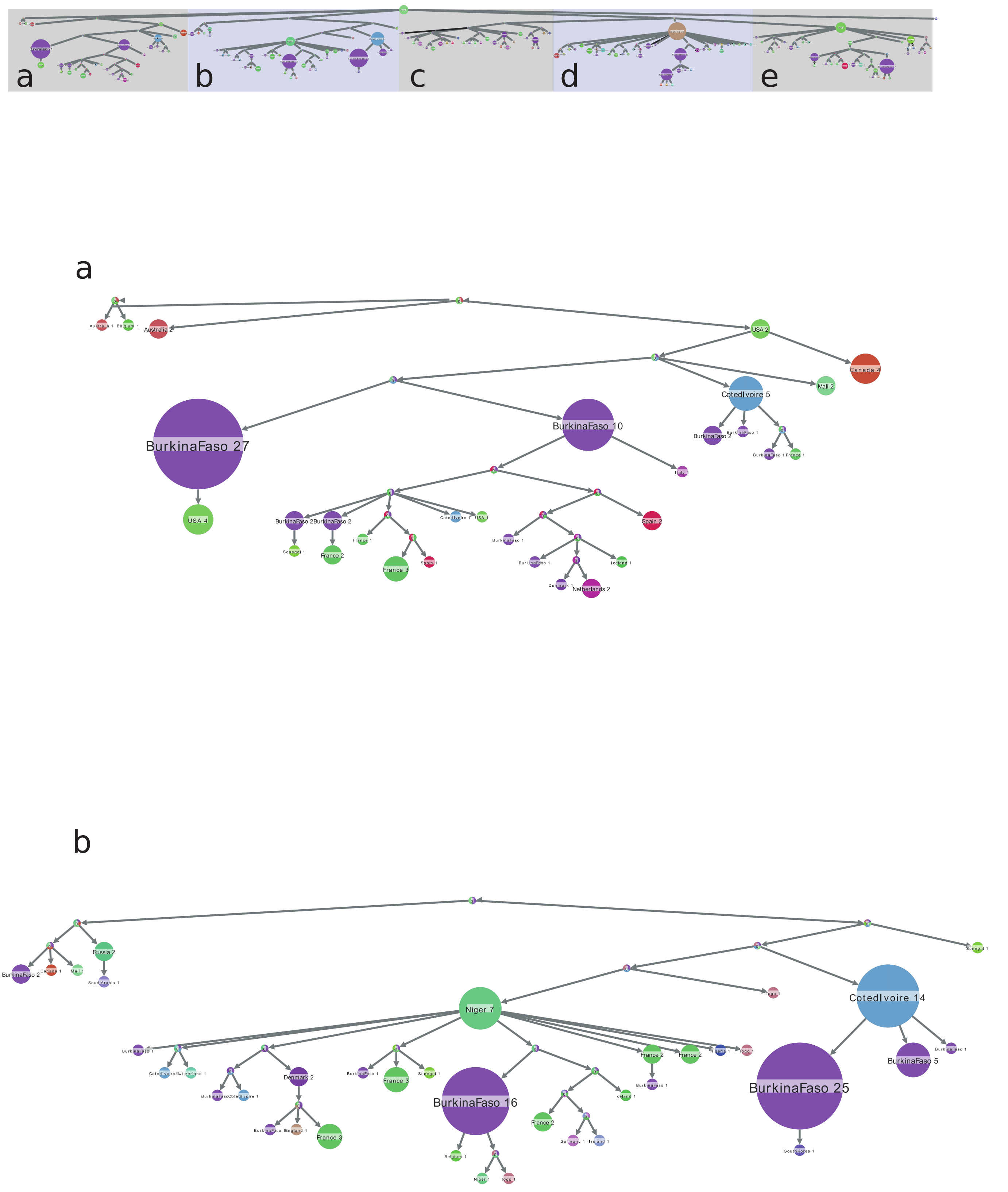
a
b
c
d
e
Australia 2
USA 2
Canada 4
Mali 2
CotedIvoire 5
BurkinaFaso 27
BurkinaFaso 10
BurkinaFaso 2
USA 4
Spain 2
France 2
France 3
Netherlands 2
Russia 2
BurkinaFaso 2
Niger 7
CotedIvoire 14
BurkinaFaso 16
BurkinaFaso 25
BurkinaFaso 5
Denmark 2
France 3
France 2
France 2
France 2
France 3
